# Electrothermal Actuator Performance Analysis via the Moving Least Square Method

**DOI:** 10.3390/mi17050542

**Published:** 2026-04-28

**Authors:** Yuanhu Gu, Jiansheng Liu, Zhangping You, Longfei Wu, Hao Chen

**Affiliations:** 1 School of Artificial Intelligence, Lishui University, Lishui 323000, China; 18921884431@189.com; 2 State Key Laboratory of Fluid Power and Mechatronic Systems, Zhejiang University, Hangzhou 310027, China; 3 School of Engineering, Lishui University, Lishui 323000, China; liujiansheng@ncu.edu.cn (J.L.); youzping@163.com (Z.Y.); zjwlf87@163.com (L.W.); 4 Zhejiang Key Laboratory of Aviation Metal Pipe Bending Technology and Equipment, Lishui 323000, China

**Keywords:** moving least squares method, electrothermal actuator, meshless, thermal measurement

## Abstract

This paper presents a case study demonstrating the use of the moving least square method (MLS) for modeling the mechanical response of an electrothermal microactuator. Under the MLS framework, the governing equations for heat transfer and structural mechanics are discretized across the computational domain. The resulting discrete electrothermal system is solved accurately through an incremental load and Newton–Raphson iterative method to determine the temperature field. Subsequently, the displacement field is obtained by solving the discrete mechanical equation, which includes contributions from natural boundary conditions. Convergence of the temperature solution is rigorously evaluated across different iterative schemes. The accuracy of the MLS solutions is validated against experimental temperature data and finite element method (FEM) simulations. Results indicate that the temperature distribution obtained from the MLS aligns well with both experimental and FEM results, even under idealized boundary conditions. Additionally, a similarly favorable comparison is observed between the displacement fields predicted by the MLS, polynomial point interpolation collocation method (PPCM), and FEM.

## 1. Introduction

Owing to advantages including compact structure, high process integration, low-voltage actuation, and large stroke output [1,2,3,4], electrothermal actuators have long maintained a leading position at the research frontier of the micro/nano manipulation field [5,6]. Their operating principle is rooted in the Joule heating effect: when an electric current passes through the structural layer, Joule heat is generated, inducing thermal expansion of the material and thus converting electrical energy into mechanical displacement. Benefiting from their typical V-shaped or U-shaped beam structures, thermal stress can be concentrated and released in a specific direction, enabling efficient and controllable linear or arc motion. As the material section expands from traditional nickel-based [7,8] and silicon-based [9] materials to phase change materials (PCMs) [10,11] and 304 stainless steel [12,13], both material and process costs have decreased significantly, accompanied by enhanced design flexibility. These emerging materials offer a broader tuning range in thermal conductivity, electrical resistivity, and mechanical strength, which enables more flexible multi-field matching of the actuators and allows them to adapt to diverse application scenarios ranging from high-frequency response to large-load output. Meanwhile, the diversification of materials has further relaxed constraints on process conditions, facilitating low-cost and high-volume manufacturing. Consequently, the application boundaries of electrothermal actuators have been continuously extended.

However, the precise motion control of micro-electrothermal actuators is severely constrained by complex microscale heat exchange with the external environment, which constitutes a primary bottleneck for their deployment in high-precision applications. Consequently, the development of accurate multi-physics coupling models and efficient computational methods has emerged as a critical research priority. Thus, the sustained and in-depth investigations have been conducted over the past decade. By employing Fourier series expansion, Zhang et al. [14] obtained the solution to the partial differential equation (PDE) describing heat transfer in the V-shaped electrothermal actuator. Chen et al. [15] predicted the steady-state behavior of an electrothermally actuated and initially curved actuator with the meshless generalized finite difference method (GFDM). By incorporating the effects of end effectors, Zhu et al. [16] introduced a combined scheme featuring the Crank–Nicolson finite difference method and the finite element method. Ma et al. [4] calculated the temperature oscillations and thermally induced bending moment based on the finite-element analysis model and Euler–Bernoulli beam theory with tension. In addition, the model solution of microactuators has also gradually introduced a series of emerging meshless computational methods. For example, Lao et al. [17] introduced a framework that integrates Physics-Informed Neural Networks (PINNs), a data-driven method fusing physical principles, with Deep Reinforcement Learning (DRL) to enable high-precision vibration control of piezoelectric cantilever beams.

Because of the operational motion and structural design, large deformation and high-order topological change widely exist in the electrothermal actuator. Traditional FEM is prone to limitations imposed by mesh distortion. In contrast, the meshless method owes independence from mesh topology and high-order continuity of shape function. Therefore, it is more suitable for the precise model and numerical computation of electrothermal actuators. Based on their solution formulation, meshless methods are generally categorized into two classes: strong-form and weak-form approaches. Weak-form methods [18,19], rooted in Galerkin formulations or the local radial point interpolation method (LRPIM), often present significant implementation complexity. This complexity primarily arises from the requirement for numerical integration over a background mesh, as well as the intricate handling of essential boundary conditions, which stems from the absence of interpolating shape functions. In contrast, strong-form methods offer advantages in terms of reduced computational cost and simplified implementation. However, they exhibit greater sensitivity to irregular node distributions and may encounter challenges related to numerical stability. Here, a comparison of some different typical meshless methods is made in Table 1. From the table, while the computational accuracy of MLS is typically moderate, its computational efficiency is notably high, attributed to the straightforward construction of shape functions and the direct discretization performed at nodes. Compared to GFDM and RPIM, MLS strikes a favorable balance by delivering greater accuracy than the former and requiring simpler implementation than the latter. Also unlike FEM, MLS is truly meshless, avoiding element connectivity and Jacobian mapping, thus eliminating mesh distortion issues when simulating large displacement electrothermal actuators. MLS also inherently provides high-order continuous shape functions, enabling direct computation of smooth temperature gradients and thermal stress fields without postprocessing smoothing. Moreover, it allows easy adaptive refinement by local refinement of node influence domains without remeshing, which is essential for resolving steep thermal gradients near electrical contacts or narrow thermal arms.

This work focuses on developing a robust and efficient numerical scheme using the MLS for the electrothermal and thermomechanical analysis of a V-shaped microactuator. The organization of the paper is as follows. Section 2 describes the fundamental principles of the MLS and presents a flowchart for actuator analysis. Section 3 formulates the discretized equations by coupling the MLS with incremental load and the Newton–Raphson iterative method, based on a previous electrothermal coupling model. Section 4 estimates actuator deflection under a thermoelastic framework using the MLS. Section 5 compares the thermal and mechanical results obtained from the MLS, FEM, PPCM, and experimental measurements and evaluates the accuracy and convergence of the MLS. Section 6 concludes the paper and proposes directions for future research.

## 2. Moving Least Squares Method

For the sake of clarity, the shape function based on the moving least squares method will be derived in matrix form. Suppose that *N* discrete points exist in a planar problem domain; *N^l^* points will be found in the local support domain of a given point **x***_I_* (*x_I_*, *y_I_*). Thus, the field variable vector **u** can be expressed by the expansion of polynomials.

(1) u=Qa

where **a** is the vector of undetermined coefficients,

(2) $$u_{s}={\left[u_{1},u_{2},\ldots ,u_{N^{l}}\right]}^{T}$$

Here, complete 2nd-order polynomials are served as basis functions to construct **Q**.

(3) $$Q=\left[\begin{array}{llllll} 1 & x_{1} & y_{1} & x_{1}^{2} & y_{1}^{2} & x_{1}y_{1}\\ 1 & x_{2} & y_{2} & x_{2}^{2} & y_{2}^{2} & x_{2}y_{2}\\ \vdots & \vdots & \vdots & \vdots & \vdots & \vdots \\ 1 & x_{N^{l}} & y_{N^{l}} & x_{N^{l}}^{2} & y_{N^{l}}^{2} & x_{N^{l}}y_{N^{l}} \end{array}\right]$$

Further, a local residual function ∏ can be expressed as

(4) $$\prod ={\left(Qa-\bar{u}\right)}^{T}W(Qa-\bar{u})$$

where $\bar{u}$ is the exact solution and Qa can be taken as fitted approximation,

(5) $$W=diag(w_{1I},\ldots w_{jI},\ldots w_{N^{l}I})$$

The *w_jI_* is obtained by the weighting function, which can be found in Table 1. The value of the weighting function used in the MLS should decrease with the distance between points **x***_I_* and **x***_j_* growing. For minimizing residual function ∏, we have

(6) $$\frac{\partial \prod }{\partial a}=2{\left(Qa-\bar{u}\right)}^{T}WQ=0$$

Further,

(7) $$a=G\bar{u},\;\;G={\left(Q^{T}WQ\right)}^{-1}Q^{T}W$$

Substituting Equation (7) into Equation (1), the shape function **N**(x,y) can be written as

(8) $$N=bG,\;\;b=[\begin{array}{cccccc} 1 & x & y & x^{2} & y^{2} & xy \end{array}]$$

**Figure 1:**
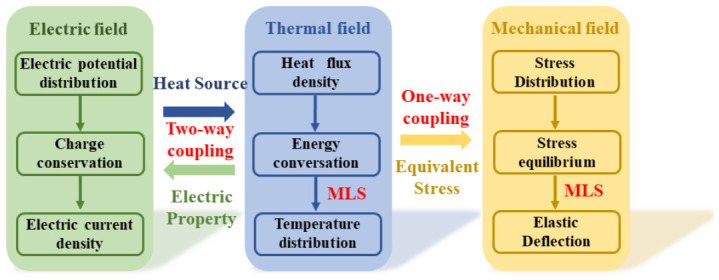
Flowchart using the MLS to predict the performance of electrothermal actuator.

In the equation, G, with 6 × *N^l^*, is a constant matrix that is merely determined by point **x***_I_*. In the planar problem, the high-order derivative terms related to the spatial domain can also be replaced by the corresponding derivative of the shape function. The brief theoretical modeling and computational analysis process on the electrothermal actuator made of polysiliconare first outlined in Figure 1. The entire computational framework, including meshfree discretization, assembly of system matrices, and iterative solvers, was implemented and validated in the MATLAB 2022a environment.

## 3. Electrothermal Analysis

As shown in Figure 1, a two-way coupling exists between electric and thermal fields. The Joule heat generated in the electric field is a stable input heat source for the thermal field. When the actuator is heated, the electric property parameters are changed and exert a significant impact on the electric potential distribution. In order to facilitate electrothermal analysis, the V-shaped actuator is regarded as a uniform beam with a constant cross-section. So this analysis can be conducted on a 1D problem domain. According to our previous study [15], the energy conservation differential equation in the thermal field used to depict the coupling relationship can be derived as

(9) $$k(T)\frac{\partial^{2}T}{\partial x^{2}}-(k_{v}\frac{w+2h}{wh}+\frac{S}{h})T+J(T)\rho (T)=0$$

where thermal conductivity *k* = 210,658(*T* − 30)^−1.2747^, width *w* and thickness *h* of the actuator are 42 and 100 μm, electrical resistivity *ρ* = 0.0002(1 + 0.003(*T* − 30)), the parameters of the heat dissipate rate *k_v_* and *S* are 70 and 0.1(*T* − 30) W·m^−2^·°C^−1^. Because of the uniform beam, the electric current density *J*(*T*), $U_{0}/\int_{0}^{2l}\rho (T)dx$, can be calculated by Ohm’s law. *U*_0_ is the voltage. At both ends of the beam, the temperatures are forced to be the room temperature. The 2nd-order polynomial is adopted in the MLS, and the vector **b** in Equation (8) can be expressed as

(10) $$b=[\begin{array}{ccc} 1 & x & x^{2} \end{array}]$$

Then matrix G is 3 × *N^l^*. For the Equation (9), its discretization form at interior point *x_I_* should satisfy

(11) $$K^{I}T^{I}=p^{I}$$

where

(12) $$K^{I}=k(T_{I})N_{I,xx}-(k_{v}\frac{w+2h}{wh}+\frac{S}{h})N_{I}$$

and

(13) $$p^{I}=-U_{0}^{2}\rho (T_{I})/(\frac{2l}{N}\sum_{j=1}^{N-1}\rho (T(x_{j})){)}^{2}$$

where $T_{I}$ denotes the temperature at node *x_I_*. The line integral of resistivity in *J*(*T*) is converted into a summation via discretization for approximate calculation. **N***_I_* and **N***_I,xx_* denote the shape function and its 2nd-order derivative at the *x_I_*, respectively.

(14) $$N_{I}=[\begin{array}{ccc} 1 & x_{I} & x_{I}^{2} \end{array}]G$$

(15) $$N_{I,xx}=[\begin{array}{ccc} 0 & 0 & 2 \end{array}]G$$

Thus, the local linear Equation (11) needs to be incorporated into the total matrix Equation (16)

(16) KT=P

**Figure 2:**
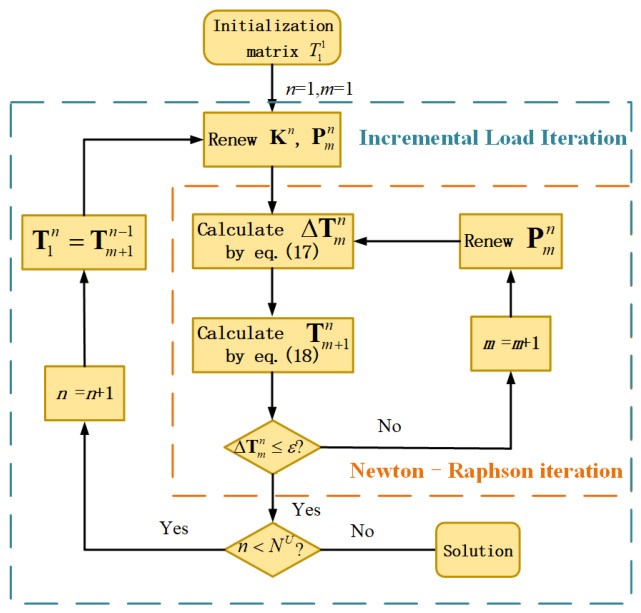
Iteration computation on the electrothermal analysis by the MLS.

The global stiffness matrix **K** is of size *N^l^* × *N^l^*, while the vectors **T** and **P** are of size *N^l^* × 1. For any element $K_{1,j}^{I}$ in the $K^{I}$, *I* denotes the global index of the point *x_I_* in the whole computational domain, consider a node *x_j_* in the support domain of node *x_I_* with local index j, which maps to global index *J*. Then, the local stiffness contribution $K_{1,j}^{I}$ corresponding to these nodes is added to the global stiffness matrix **K** at position (*I*, *J*). Likewise, the local vector contributions $T_{j}^{I}$ and *p_I_* are added to the global vectors T at position *J* and P at position *I*, respectively. Upon assembly, essential boundary conditions are enforced at the two boundary nodes. The strong temperature-dependent nonlinearity of *ρ* and *k* means that Equation (16) must be solved using a hybrid scheme involving both incremental load iteration and the Newton–Raphson method (I-NR). $T_{m}^{n}$ refers to the temperature value at the *m*th Newton-Raphson iterative step, which occurs during the *n*th increment with an input voltage of $\sqrt{U_{0}^{2}/N^{U}}$. Here, *N^U^* denotes the total number of incremental load iterations. The iterative computation procedure is illustrated in Figure 2, and the detailed steps are described as follows:

(i) Based on temperature $T_{m}^{n}$, the stiffness matrix $K^{n}$ and load vector $P_{m}^{n}$ are obtained. Further, the corrected temperature quantity $\Delta T_{m}^{n}$ is calculated by the Newton–Raphson method.

(17) $$\Delta T_{m}^{n}={\left(K^{n}\right)}^{-1}(K^{n}T_{m}^{n}-P_{m}^{n})$$

(ii) The temperature $T_{m+1}^{n}$ at the (*m* + 1)th Newton-Raphson iterative step

(18) $$T_{m+1}^{n}=T_{m}^{n}+\Delta T_{m}^{n}$$

(iii) Termination condition judgment in the inner loop: if the convergence condition, $\Delta T_{m}^{n}\leq \varepsilon$, is satisfied, go to the next step. Otherwise, return to the step (i). Here, ε is the error tolerance in the Newton–Raphson iteration.

(iv) Termination condition judgment in the outer loop: if $n<N^{U}$, the calculation is stopped. Otherwise, $T_{1}^{n+1}=T_{m+1}^{n}$ and go back to the step (i).

As is shown in Figure 2, the Newton-Raphson iteration is conducted in each incremental load iteration, which further improves the accuracy of the solution. Moreover, given the complicated matrix assembly in Equation (11) and inverse computation in Equation (17), the stiffness matrix **K** is not renewed in the inner loop iteration.

## 4. Thermomechanical Analysis

By incorporating the isotropic thermal expansion coefficient with the temperature distribution derived from the preceding analysis, the thermal strain profile can subsequently be determined using the discrete domain. Accordingly, the steady-state thermoelastic equation is expressed as follows:

(19) $$\left\{\begin{array}{c} \frac{E}{1-\mu^{2}}(\frac{\partial^{2}u}{\partial x^{2}}+\frac{1-\mu }{2}\frac{\partial^{2}u}{\partial y^{2}}+\frac{1+\mu }{2}\frac{\partial^{2}v}{\partial x\partial y}-\frac{\partial \varepsilon_{x}^{t}}{\partial x}-\mu \frac{\partial \varepsilon_{y}^{t}}{\partial x})=0\\ \frac{E}{1-\mu^{2}}(\frac{\partial^{2}v}{\partial y^{2}}+\frac{1-\mu }{2}\frac{\partial^{2}v}{\partial x^{2}}+\frac{1+\mu }{2}\frac{\partial^{2}u}{\partial x\partial y}-\mu \frac{\partial \varepsilon_{x}^{t}}{\partial y}-\frac{\partial \varepsilon_{y}^{t}}{\partial y})=0 \end{array}\right.$$

Here *E* and *μ* represent the Young’s modulus and Passion’s ratio, respectively, while *u* and *v* denote the displacement components in the *x-* and *y*-directions. The thermal strain in the x-direction is expressed as *ε_x_^t^* = *α*(*T*) × *T*, where the thermal expansion coefficient *α* varies with the temperature according to *α =* −4 × 10^−12^*T*^2^ + 8 × 10^−9^*T* + 4 × 10^−7^. The natural boundary condition is given by:

(20) $$\left\{\begin{array}{c} \frac{E}{1-\mu^{2}}(n_{x}\frac{\partial u}{\partial x}+\mu n_{x}\frac{\partial v}{\partial y}+\frac{1-\mu }{2}n_{y}(\frac{\partial u}{\partial y}+\frac{\partial v}{\partial x})+n_{x}(\varepsilon_{x}^{t}+\mu \varepsilon_{y}^{t}))=0\\ \frac{E}{1-\mu^{2}}(n_{y}\frac{\partial v}{\partial y}+\mu n_{y}\frac{\partial u}{\partial x}+\frac{1-\mu }{2}n_{x}(\frac{\partial u}{\partial y}+\frac{\partial v}{\partial x})+n_{y}(\mu \varepsilon_{x}^{t}+\varepsilon_{y}^{t}))=0 \end{array}\right.$$

Here, the quantities *n_x_* and *n_y_* are the *x* and *y* components of the normal vector. Moreover, the actuator ends are constrained by the essential boundary condition: *u* = 0 and *v* = 0. For the Equation (11), the elements of stiffness matrix **K***^I^* and load vector *p^I^* are determined by the position (*x_I_*, *y_I_*) of the discretization point **x***_I_*.

Case I: If the discretization point **x***_I_* is situated in the internal region of the computational domain, the expressions for **K***^I^* and *p^I^* are obtained from Equation (21). In this case, **K***^I^* has dimensions 2 × 2*N^l^*.

(21) $$\left\{\begin{array}{c} K_{1,2*j-1}^{I}=\frac{E}{1-\mu^{2}}(N_{I,xx}+\frac{1-\mu }{2}N_{I,yy})\\ K_{1,2*j}^{I}=\frac{E}{2(1-\mu )}N_{I,xy} \end{array}\right.$$

(22) $$\left\{\begin{array}{c} K_{2,2*j-1}^{I}=\frac{E}{2(1-\mu )}N_{I,xy}\\ K_{2,2*j}^{I}=\frac{E}{1-\mu^{2}}(N_{I,yy}+\frac{1-\mu }{2}N_{I,xx}) \end{array}\right.$$

where **N***_I,xx_*, **N***_I,xy_* and **N***_I,yy_* can also be obtained based on Equation (8), in which partial 3rd order polynomials are added in order to improve the computational accuracy. The vector b can be expressed as

(23) $$b=\left[\begin{array}{cccccccc} 1 & x & y & x^{2} & y^{2} & xy & x^{2}y & xy^{2} \end{array}\right]$$

Thus, the **N***_I,xy_* is derived as

(24) $$N_{I,xy}=[\begin{array}{cccccccc} 0 & 0 & 0 & 0 & 0 & 1 & 2x & 2y \end{array}]G$$

and

(25) $$\left\{\begin{array}{c} p_{1}^{I}=\frac{E}{1-\mu^{2}}(\varepsilon_{Ix,x}^{t}+\mu \varepsilon_{Iy,x}^{t})\\ p_{2}^{I}=\frac{E}{1-\mu^{2}}(\mu \varepsilon_{Ix,y}^{t}+\varepsilon_{Iy,y}^{t}) \end{array}\right.$$

where $\varepsilon_{Ix,x}^{t}$ and $\varepsilon_{Iy,x}^{t}$ are the 1st partial derivatives of the *x*-direction and *y*-directional thermal strain with respect to *x* at point **x***_I_*. They are evaluated from the temperature distribution by means of the central finite difference method. Consistent with the 1D model used in the electrothermal analysis, $\varepsilon_{Ix,y}^{t}$ and $\varepsilon_{Iy,y}^{t}$ are both assumed to be zero.

Case II: If the point **x***_I_* is at the natural boundary, the **K***^I^* and *p^I^* should be derived based on the Equation (22).

(26) $$\left\{\begin{array}{c} K_{1,2*j-1}^{I}=\frac{E}{1-\mu^{2}}(n_{Ix}N_{I,x}+\frac{1-\mu }{2}n_{Iy}N_{I,y})\\ K_{1,2*j}^{I}=\frac{E}{1-\mu^{2}}(\mu n_{Ix}N_{I,y}+\frac{1-\mu }{2}n_{Iy}N_{I,x}) \end{array}\right.$$

(27) $$\left\{\begin{array}{c} K_{2,2*j-1}^{I}=\frac{E}{1-\mu^{2}}(\mu n_{Iy}N_{I,x}+\frac{1-\mu }{2}n_{Ix}N_{I,y})\\ K_{2,2*j}^{I}=\frac{E}{1-\mu^{2}}(n_{Iy}N_{I,y}+\frac{1-\mu }{2}n_{Ix}N_{I,x}) \end{array}\right.$$

where **N***_I,x_*, is

(28) $$N_{I,x}=[\begin{array}{cccccccc} 0 & 1 & 0 & 2x & 0 & y & 2xy & y^{2} \end{array}]G$$

Other first-order derivatives of the shape function vector can be obtained by the same method, according to Equation (8).

(29) $$\left\{\begin{array}{c} p_{1}^{I}=\frac{E}{1-\mu^{2}}n_{Ix}(\varepsilon_{Ix}^{t}+\mu \varepsilon_{Iy}^{t})\\ p_{2}^{I}=\frac{E}{1-\mu^{2}}n_{Iy}(\mu \varepsilon_{Ix}^{t}+\varepsilon_{Iy}^{t}) \end{array}\right.$$

where *n_Ix_* and *n_Iy_* are components of the normal vector in the *x* and *y*-directions at the point **x***_I_*.

Case III: Otherwise, the point **x***_I_* is at the Dirichlet boundary. To meet the coercive boundary condition, matrices **K***^I^* and *p^I^* are directly expressed as.

(30) $$K_{1,1}^{I}=K_{2,2}^{I}=1$$

and

(31) $$p_{1}^{I}=p_{2}^{I}=0$$

where the remaining entries of matrix **K***^I^* are 0. Subsequently, Equations (20)–(22), (25)–(27) and (29)–(31) need to be assembled into a global matrix equation of the form given in Equation (12). In this global system, **K** is a 2*N^l^* × 2*N^l^* matrix, and *P* is a 2*N^l^* × 1 vector. Considering an element $K_{1,2*j-1}^{I}$, where *I* is the global index and *j* the local index, and *J* denotes the global index of **x**_j_ in the problem domain, the assembly proceeds as follows: $K_{1,2*j-1}^{I}$ is inserted into the row (2*I* − 1), column (2*J* − 1) of **K**; $K_{2,2*j-1}^{I}$ goes into row (2*I*) and column (2*J* − 1); and the load terms $p_{1}^{I}$ and $p_{2}^{I}$ are placed in rows (2*I* − 1) and (2*I*) row of **P**, respectively. This construction yields a global matrix equation that can be efficiently solved by the direct iteration method.

## 5. Numerical Results and Discussion

- Thermal measurement

**Figure 3:**
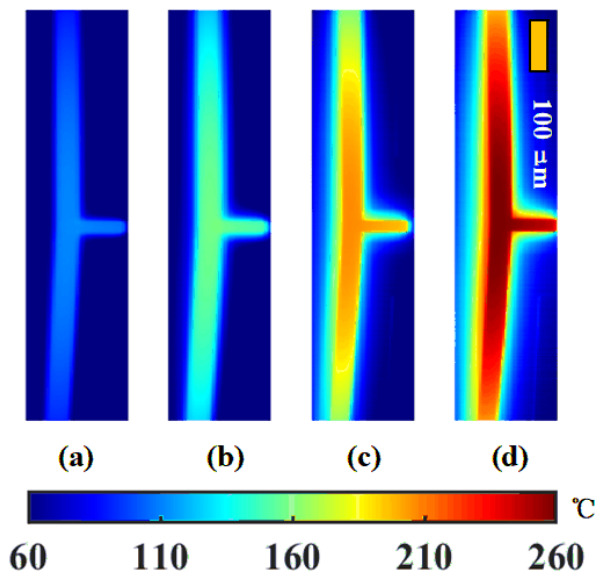
Experimental temperature distributions under different voltages (**a**) 4 V, (**b**) 5 V, (**c**) 6 V, (**d**) 7 V.

To investigate thermal behavior, an infrared thermal microscope system was used to map the temperature profile of the V-shaped actuator. The need for high-resolution imaging restricted the observable area, focusing on a narrow field of view. Previous studies have highlighted the critical role of the high-temperature zone in the actuator’s mid-section on its overall performance. Hence, the experimental strategy prioritized capturing thermal images of this region. Through integration of actuator scale and image processing, the pixel size was calibrated to roughly 3.75 μm. Figure 3 illustrates the temperature profiles over a region roughly 1470 μm in length of the actuator under different voltage levels, revealing a pattern of symmetrical decrease from the midpoint outward. In the figure, the temperature increases rapidly as the voltage increases uniformly. Under a 7 V load, the central area exhibited a maximum temperature near 262 °C. The observed temperature elevation relative to ambient suggests the presence of thermal radiation effects.

B. Thermal prediction

**Figure 4:**
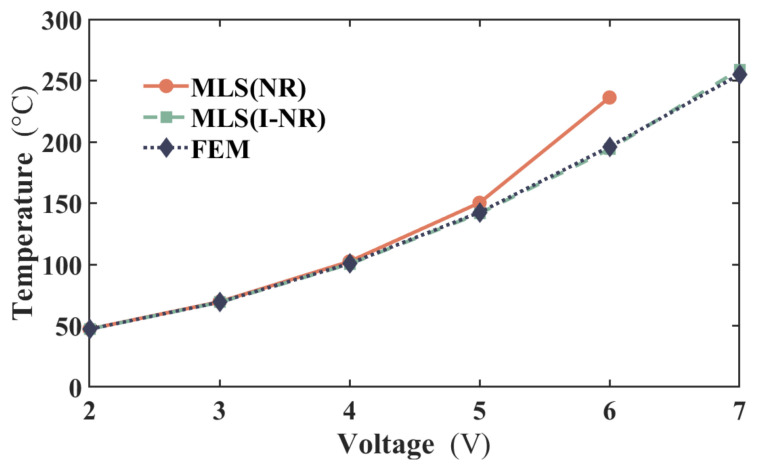
Middle temperatures of the actuator with different iterative methods and FEM under different voltages.

To assess the performance of the Newton-Raphson iteration incorporated into the incremental load iteration method (I-NR) employed in the MLS, the basic Newton-Raphson iteration method (NR) is also adopted to solve Equation (10), with FEM results serving as reference values. As shown in Figure 4, the temperature discrepancy arising from the NR method increases with applied voltage, reaching approximately 40 °C at 6 V. When a voltage of 7 V is applied, the computation fails to converge with the NR method. In contrast, the I-NR method always exhibits significantly improved convergence within the PPCM framework, yielding temperature differences below 4 °C compared to FEM across all voltage levels. Thus, the combination of the MLS and I-NR is preferable for achieving accurate results in this work. Such behavior stems from the strongly nonlinear characteristics of Equation (9), where thermal conductivity rises with temperature while electrical resistivity follows an inverse trend.

**Figure 5:**
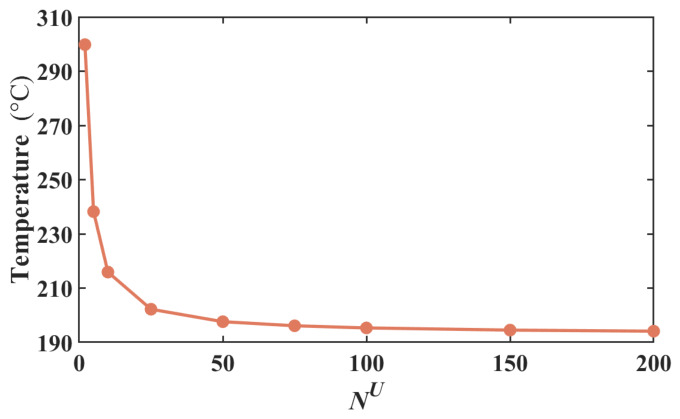
Middle temperatures varying with incremental load iteration *N^U^* under 6 V.

**Figure 6:**
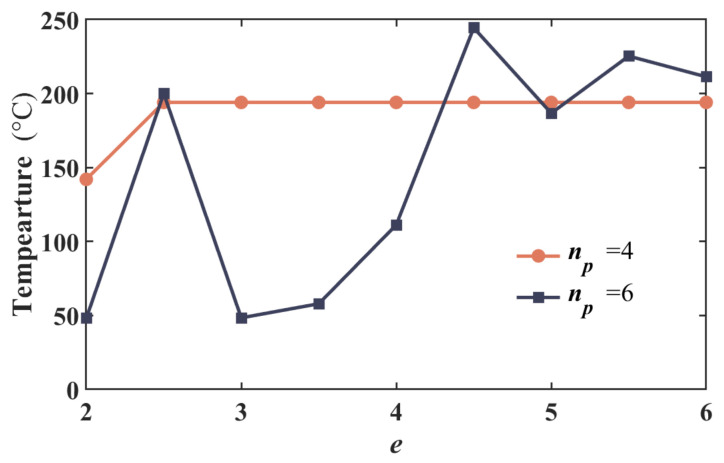
Middle temperatures varying with the length of the support domain under different polynomial orders.

To further investigate the computational stability of the I-NR-based MLS, a convergence test was conducted. Three distinct weighting functions are employed within the MLS framework to solve the governing equation, and the resulting maximum temperatures at 6 V are presented in Table 2. In the weighting functions, $\bar{r}$ denotes the normalized radius, which is the ratio of the actual distance to the radius of the support domain. As illustrated in the table, the MLS exhibits insensitivity to the choice of weighting function, with all cases demonstrating excellent convergence behavior. The convergence test of the number of incremental load iterations *N*^U^ is conducted and presented in Figure 5. In the figure, the temperature tends to be stable with the number of steps increasing. The recommended value of *N*^U^ is 100, at which point the temperature shows negligible difference. The relationship between the convergence range *e*, the ratio of the support domain radius to the meshless node spacing, and polynomial order n*_p_* is shown in Table 3. Polynomials of the 3rd to 5th order, adopted in the MLS, are suitable for electrothermal analysis. When a 6th-order polynomial is employed, the temperature calculation results exhibit a gradual divergence, which can be found in Figure 6.

**Figure 7:**
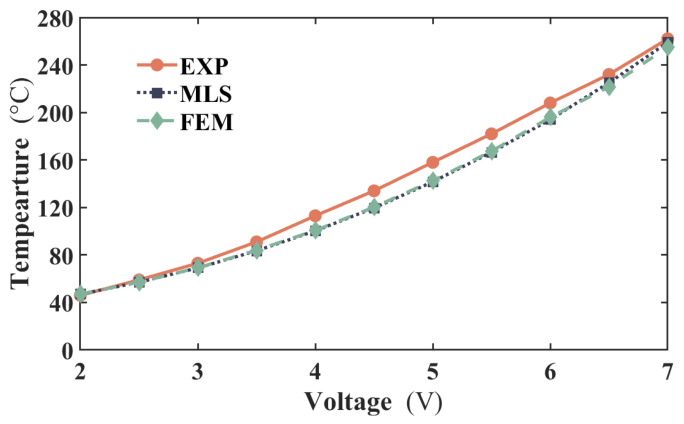
Middle temperatures comparison between the experiment, MLS, and FEM from 2–7 V.

Figure 7 compares the steady-state temperatures obtained from the MLS, FEM, and experiments at various applied voltages, with all temperature values extracted from the central region of the actuator. As illustrated, the temperature curve predicted by the MLS exhibits strong agreement with that from the FEM. Furthermore, it closely aligns with the experimental data, with a maximum temperature discrepancy of approximately 11 °C. The root-mean-square (RMS) temperature errors and maximum relative errors for both methods are computed. The RMS errors are 10.4 °C for MLS and 10.2 °C for FEM, while the maximum relative temperature errors are 10.3% and 9.8%, respectively. These results demonstrate that MLS and FEM achieve nearly identical overall accuracy, with differences of only 0.2 °C in RMS error and 0.5% in maximum relative error. The slightly higher errors in MLS are within an acceptable range (<5% difference) and do not compromise its effectiveness for electrothermal actuator analysis. This further supports the validity of MLS as a reliable meshfree alternative to FEM. For a more detailed analysis, temperatures at several points along the actuator length were extracted from the thermal images.

**Figure 8:**
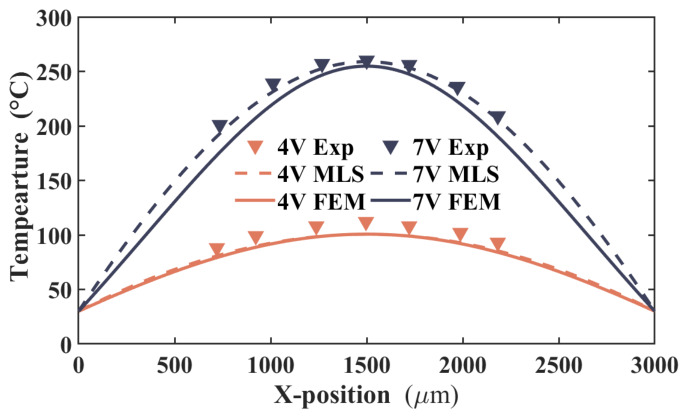
Comparison of the temperature distribution between the experiment, MLS, and FEM at 4 and 7 V.

Figure 8 presents a comparison of the temperature profiles obtained from the MLS and FEM. Under applied voltages of 4 V and 7 V, the MLS profile correlates well with the FEM results, showing a maximum deviation of roughly 17 °C near the quarter-span points at 7 V in which the largest local temperature gradient exists. The MLS also shows less favorable agreement when compared against the experimental temperature profile. In this case, the temperature difference progressively increases from the center toward both ends of the actuator. At 7 V, the maximum error is estimated to reach approximately 11 °C. This discrepancy can primarily be attributed to the use of a Dirichlet boundary condition at both ends of the actuator in the simulation, which does not accurately reflect the actual experimental conditions. In addition, at 4 V under moderate heating, MLS and FEM yield very close accuracy: RMS temperature errors are 9.2 and 9.6, while the maximum relative errors are 10.1% and 10.8%. At 7 V under higher heating, MLS significantly outperforms FEM: RMS errors are 5.9 and 15.9, while maximum relative errors are 3.71% and 12.9%. The RMS error of MLS is approximately 63% lower than that of FEM, and the maximum relative error is reduced by about 71%. These results demonstrate that the advantage of the MLS becomes increasingly pronounced under more severe thermal loading, specifically at higher temperatures and steeper thermal gradients. At 7 V, MLS achieves superior accuracy primarily due to its meshless formulation and high-order continuously differentiable shape functions, which inherently eliminate mesh distortion and enable robust representation of strongly nonlinear temperature fields. In contrast, FEM exhibits reduced fidelity in high-gradient regions, which owes to compromised local resolution and solution smoothness because of its limitations in interpolation order.

C. Mechanical prediction

**Figure 9:**
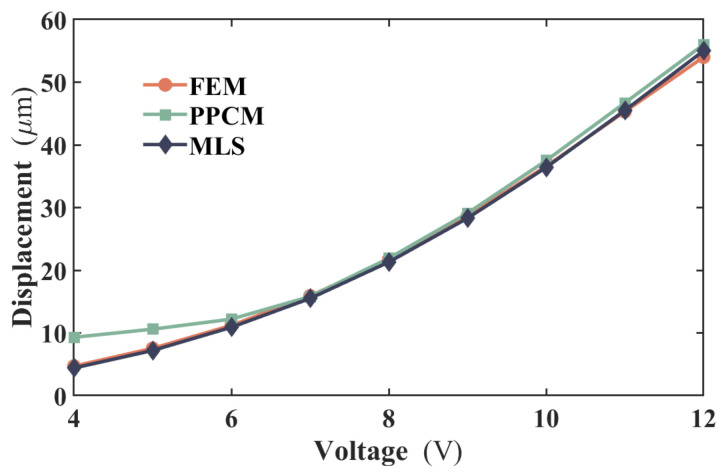
Maximum displacement of the actuator from different computational methods loaded with different voltages.

The computational stability and efficiency of the MLS for thermomechanical analysis were evaluated. The weighting function W3 in Table 1 and support domain radius of d = 101 μm are employed, respectively, where *c* is also determined to be 2. Figure 9 presents a comparison of the displacements at the actuator’s central region—where the maximum displacement occurs—as computed by the MLS and FEM under various applied voltages. The results calculated by PPCM in our previous study [25] are also added to make a comparison. From the figure, the displacement curve obtained from the MLS always exhibits generally good agreement with that from the FEM. At an applied voltage of 12 V, the displacement error is merely about 1 μm. However, noticeable discrepancies are observed at lower voltages between PPCM and FEM. MLS performs better in the prediction of displacement for electrothermal actuators than PPCM. Given its satisfactory performance in mechanical prediction, the MLS is considered suitable for solving the thermomechanical model of the actuator.

## 6. Conclusions

This work demonstrates the effective application of the moving least squares method to the coupled electro-thermo-mechanical analysis of a V-shaped microactuator. For the electrothermal part, the RMS middle temperature error between the MLS and the experiment is about 10.4 °C when different voltages are applied to the actuator. At 7 V under higher heating, MLS significantly outperforms FEM; the RMS temperature distribution errors are 5.9 and 15.9 °C. The RMS error of MLS is approximately 63% lower than that of FEM. The simulation and experimental results verify the combination of the incremental load with the Newton–Raphson method adopted in MLS for the high nonlinearity in the electrothermal analysis. Thus, when the actuator is loaded with higher operating voltage and the thermal gradient is significant, the MLS method is recommended for simulation to avoid mesh distortion and interpolation errors inherent in FEM. For the thermomechanical part, with the implementation of the MLS, the mechanical characteristics are also perfectly predicted. The maximum displacement error is no more than 1 μm between the MLS and FEM when a voltage of 12 V is loaded. Collectively, these outcomes suggest that the MLS-based approach offers an efficient, stable, and accurate numerical framework for multiphysics problems. Additionally, because the method requires only a discrete set of nodes in the solution process, it qualifies as a truly meshless technique. This capability makes it a promising candidate for future applications in topology optimization involving multiphysics systems.

## Figures and Tables

**Table 1:** Comparison and analysis on different meshless methods.

Method	Shape Function	Integration	Accuracy	Efficiency
RPIM [20]	Background mesh + Radial basis function	Background mesh	High	Low
MLPG [21]	Hybrid method (RPIM/MLS)	Local domain	High	Low
GFDM [22]	Local Taylor expansion + MLS	/	Middle	Middle
MLS [23]	Polynomial + MLS	/	Middle	High
PINN [24]	Neural network	/	Middle	Low

**Table 2:** Middle temperature under different weight functions adopted in the MLS.

Name	Expression	Temperature/°C
W1	$1-6{\bar{r}}^{2}+8{\bar{r}}^{3}-3{\bar{r}}^{4}$	194.1
W2	$\left\{\begin{array}{cc} 2/3-4{\bar{r}}^{2}+4{\bar{r}}^{3} & \bar{r}\leq 0.5\\ 4/3-4\bar{r}+4{\bar{r}}^{2}-4/3{\bar{r}}^{3} & 0.5<\bar{r}\leq 1 \end{array}\right.$	194.1
W3	$\exp(-{\left(\bar{r}/c\right)}^{2})$	194.1

**Table 3:** Convergence range *e* varying with the polynomial order *n_p_.*

*n_p_*		2	3	4	5	6
Convergence range	*e_l_*	/	1	2	2.6	/
	*e_u_*	/	5	7	7.7	/

## Data Availability

The original contributions presented in this study are included in the article. Further inquiries can be directed to the corresponding author.
